# How confident are medical students about making clinical decisions relying on the evidence? A cross-sectional questionnaire study

**DOI:** 10.3205/zma001292

**Published:** 2019-11-15

**Authors:** Luca Frank, Susann Hueber, Piet van der Keylen, Marco Roos

**Affiliations:** 1 Friedrich-Alexander-Universität Erlangen-Nürnberg (FAU), Allgemeinmedizinisches Institut, Erlangen, Germany

**Keywords:** medical students, evidence-based medicine, clinical decision-making, uncertainty, doctoral thesis, pictogram

## Abstract

**Objective: **Giving information and providing advice on diagnostic tests is one of the tasks physicians must carry out personally. To do so, they must evaluate the evidence and integrate their findings into everyday practice. Clinical decisions should be based on evidence. How well current medical education prepares for such evidence-based clinical decision-making is largely unclear. Therefore, it was examined how confident medical students are in clinical decision-making based on evidence using epidemiological data. It was examined whether the decision-making confidence increases the higher the semester. Further questions were whether scientifically active medical students show higher decision-making confidence and whether the representation of figures as pictograms rather than tables positively influences the decision-making confidence.

**Methods: **An online survey of the medical students of the Friedrich-Alexander-University Erlangen-Nürnberg was carried out. Respondents were presented with three clinical decision-making situations in random order for evaluation in the form of screening scenarios. In each case, the decision-making confidence also had to be specified. The scenarios contained only epidemiological data on existing screening tests. For each scenario, the numbers were presented as a table or a pictogram in a random fashion. In order to avoid false confidence resulting from preconceived opinions neither the illnesses nor the screening tests were mentioned by name.

**Results: **Answers from 171 students were evaluated. Decision-making confidence in dealing with the numbers does not increase in higher semesters (*r_Pearson_*=0.018, *p*=0.41). Scientific work is not associated with a higher decision-making confidence (*t*(169)=-1.26, *p*=0.11, *d*=-0.19). Presentation as a pictogram leads to a higher decision-making confidence compared to tables (Pictogram: *M*=2.33, *SD*=1.07, Table with numbers: *M*=2.64, *SD*=1.11, *t*(511)=3.21, *p*<0.01, *d*=0.28).

**Conclusions: **Medical students from higher semesters show no higher decision-making confidence compared to medical students from lower semesters. Curricular events and scientific work, such as a doctoral thesis, do not seem to strengthen the required skills sufficiently. If evidence is presented in the form of pictograms, this seems to improve student confidence in decision-making.

## Zusammenfassung

**Zielsetzung: **Die Aufklärung und Beratung zu diagnostischen Tests gehören zu den von Ärztinnen und Ärzten höchstpersönlich zu erbringenden Leistungen. Dazu müssen sie Evidenz bewerten und deren Erkenntnisse in den Praxisalltag integrieren. Klinische Entscheidungen sollten schließlich auf Basis der Evidenz getroffen werden. Wie gut die aktuelle medizinische Ausbildung auf diese evidenzbasierte klinische Entscheidungsfindung vorbereitet, ist weitgehend unklar. Deshalb wurde untersucht, wie sicher Medizinstudierende bei der klinischen Entscheidungsfindung anhand von epidemiologischen Zahlen aus der Evidenz sind. Es wurde geprüft, ob die Entscheidungssicherheit mit höherem Fachsemester steigt. Weitere Fragestellungen waren, ob wissenschaftlich tätige Medizinstudierende dabei eine höhere Entscheidungssicherheit zeigen, und ob die Darstellung der Zahlen als Piktogramm versus Tabelle die Entscheidungssicherheit positiv beeinflusst.

**Methodik: **Es wurde eine Onlinebefragung der Medizinstudierenden der Friedrich-Alexander-Universität Erlangen-Nürnberg durchgeführt. Den Befragten wurden drei klinische Entscheidungssituationen in Form von Früherkennungs-Szenarien in zufälliger Reihenfolge zur Bewertung vorgelegt. Dabei musste jeweils auch die Entscheidungssicherheit angegeben werden. Die Szenarien enthielten ausschließlich epidemiologische Zahlen zu existierenden Früherkennungsmaßnahmen. Für jedes Szenario wurde randomisiert zugeteilt, ob die Zahlen als Tabelle oder Piktogramm dargestellt wurden. Um falsche Sicherheit durch vorgefasste Meinungen zu umgehen, wurden weder die Erkrankungen noch die Maßnahmen namentlich erwähnt.

**Ergebnisse:** Antworten von 171 Studierenden wurden ausgewertet. Die Entscheidungssicherheit im Umgang mit den Zahlen steigt nicht in höheren Fachsemestern (*r_Pearson_*=0.018, *p*=0.41). Eine wissenschaftliche Tätigkeit ist nicht mit einer höheren Entscheidungssicherheit assoziiert (*t*(169)=-1.26, *p*=0.11, *d*=-0.19). Die Darstellung als Piktogramm führt im Vergleich zur Tabelle zu einer höheren Entscheidungssicherheit (Piktogramm: *M*=2.33, *SD*=1.07, Zahlentabelle: *M*=2.64, *SD*=1.11, *t*(511)=3.21, *p*<0.01, *d*=0.28).

**Schlussfolgerung: **Medizinstudierende aus höheren Fachsemestern zeigen keine höhere Entscheidungssicherheit im Vergleich zu Medizinstudierenden aus niedrigeren Fachsemestern. Curriculare Veranstaltungen und wissenschaftliche Tätigkeit, wie eine Doktorarbeit, scheinen die dafür benötigten Kompetenzen bisher nicht ausreichend zu stärken. Wird Evidenz in Form von Piktogrammen aufbereitet, so scheint dies, die Sicherheit der Studierenden bei Entscheidungen zu verbessern.

## Introduction

Giving information and advice on diagnostic tests and treatments is one of the tasks physicians must carry out personally [1]. In order to do so, they must evaluate study results and transfer the insights into everyday practice. The German Network for Evidence-based Medicine (DNEbM) defines this as “integration of individual clinical expertise with the best possible external evidence from systematic research (modified after [2])” in the sense of evidence-based medicine (EBM) [3]. For example, epidemiological figures provide information on the benefits and risks of screening. However, the ability to interpret such numbers from studies such as mortality, morbidity, and Number-Needed-to-Screen (NNS) is often poorly taught in medical education. As part of a representative survey of Bavarian graduates of human medicine, only 30.4% of the respondents stated that they had acquired the necessary competence for scientific action, such as the evaluation of studies, during their studies [4].

There were significant gaps in scientific competence in dealing with numbers among medical students in a study of American medical students in their first year [5]. Almost one in four had problems with the simple conversion of statistical values. In the subsequent interpretation of the numbers using a treatment scenario, the proportion of students with difficulties was even greater. These problems largely remain all the way to graduation. A study of American medical students in the 4^th^ academic year showed considerable issues in dealing with statistics and critical appraisal of literature [6]. This seems to continue in post-graduate education. In a survey three-quarters of American assistant physicians in their first year of work expressed low confidence in dealing with statistics [7]. This was confirmed in a subsequent test of statistical capabilities. It also showed a decrease in skills with increasing time since graduation from medical school. Studies of students and assistant physicians at the University of Frankfurt show that both the knowledge of statistical procedures and study designs, as well as basic skills of EBM, such as definition of an answerable question, literature research and critical appraisal of the literature found, can be improved by additional seminars [8], [9]. However, the application of the evidence found to patients and clinical decision-making was not part of the studies. This is not surprising as these are still rarely part of EBM courses [10]. However, the ability to make confident clinical decisions is not a given. A study of American medical students in the 4^th^ year showed that they have problems in dealing with statistical uncertainties in clinical decision-making [11]. The majority of students therefore asked for further diagnostic tests to reach a clinical decision, even though there was already a very high probability of disease. As a result, these shortcomings seem to have a direct impact on medical practice. It is not clear whether the situation in Germany is similar to that in the United States. To date there are no studies on the confidence of German medical students in their clinical decision-making based on statistical data.

Perhaps it is also due to this issue that within the framework of the “Master Plan for Medical Studies 2020”, the teaching of scientific competences in medical training is accorded greater importance [12]. The new National Competency-based Learning Objectives Catalog in Medicine (NKLM) also emphasizes the importance of scientific competences and emphasizes them in the definition of the role of the physician as a scholar: *“As a scholar, physicians maintain and improve their professional practice through continuous, lifelong learning and through critical evaluation and application of scientific information and its sources*” [http://www.nklm.de]. The medical doctoral thesis is regularly cited as a vehicle for the acquisition of scientific competences. In an American study, preparation of a medical research paper, for example as part of a PhD, was associated with somewhat better statistical skills in assistant physicians [7]. However, the transferability of these results to Germany seems questionable given the different requirements for doctoral degrees. The Science Council has repeatedly criticized practices regarding doctorates and the scientific level of medical doctoral theses in Germany [13]. Previous surveys have shown that German medical students who completed a doctorate rate their scientific competence higher than their colleagues who have not yet completed their doctoral degree [14]. German medical students are of the opinion that their ability to carry out scientific work has improved by working on a doctorate [15]. However, they do not feel better prepared to work as a physician. Whether scientific work at medical school actually strengthens confidence in dealing with scientific evidence in clinical issues has not been investigated to date.

Additionally, one important aspect of clinical decision-making based on evidence data is the presentation of figures. A current guideline of DNEbM sets standards for evidence-based health information for patients [16]. It emphasizes that benefits and harms should ideally be presented as absolute risks. Presentation using relative risks often leads to misjudgment of benefits and harms, both in patients and physicians [17]. Furthermore, the same denominators should always be used [16]. A randomized controlled study, in which lay persons were presented with absolute risks in different formats, was able to show that different denominators have a negative influence on the understanding of figures [18]. According to the guidelines of the DNEbM, in addition graphics, in particular pictograms, can be used to represent risks better [16]. In a randomized controlled study of pictograms these not only improved the understanding of medical risks but were also found to be more helpful by lay persons [19]. A similarly structured study of students from different disciplines was also able to demonstrate an improvement in the understanding of risks by representing them as pictograms [20]. Whether these positive effects can also be transferred to medical professionals and medical students is still unclear. The present study aims to address the aspects mentioned above in more detail using German medical students.

It examined their confidence in decision-making based on epidemiological data in clinical questions using the example of screening tests. The individual questions were:

1. Does decision-making confidence in dealing with epidemiological data increase with higher semesters?
2. Do medical students who are scientifically active exhibit greater confidence in decision-making?
3. Does the way in which the epidemiological data are presented (tables versus pictograms) have an impact on decision-making confidence?

## Methods

### Study design

A cross-sectional survey was carried out by means of an online questionnaire. The questionnaire was developed at the General Medical Institute of the University Hospital Erlangen. It was created using SurveyMonkey and tested by two medical staff (AD, AS). The clearance certificate of the Ethics Commission of the Medical Faculty of the Friedrich-Alexander-University Erlangen-Nürnberg was granted November 2014 (327_14c). The students of Human Medicine of the Friedrich-Alexander-University Erlangen-Nürnberg (*n*=2,554) were invited to the online survey by email in November 2015 at the beginning of the winter semester. To increase the response rate, a reminder was sent a week later. There was no compensation for participating in the study. To exclude multiple entries, an IP lock was used.

#### Sample size

A total sample of 64 respondents is necessary in order to prove a medium effect (*r*=0.3) of the semester on decision-making confidence through bivariate correlation analysis with a one-sided α-error level of 0.05 with a power of 0.80. The same power and a error level was determined for detection of a medium effect (*d*=0.5) by means of unlinked t tests in the further analyzes. Thus, the unilateral comparison of students who were and were not scientifically active requires a group size of 51 and a total sample of 102 respondents. In order to demonstrate an influence of the choice of representation i.e. tables or pictograms, a two-sided a error level of 0.05 requires a group size of 64 and a total sample of 128 respondents.

#### Participants

287 medical students took part in the survey (response rate=11%). Of these, 171 students answered the survey completely. All respondents (*n*=171) were actual students of medicine. The properties of the sample correspond to the expected distribution of characteristics among German medical students. The proportion of female medical students of 68% almost coincided with the national proportion (61%) in the winter semester 2015/2016 [21]. The proportion of scientifically active students, especially in the higher semesters, was comparable to the results of a survey among German medical graduates in 2005 and 2009 [22]. This found that one and a half years after graduation, about 80% of graduates said they had either started or already completed a doctorate. At the time of the survey there was no EBM course on offer at the university. Only the cross-curricular subject of epidemiology and medical biometry, which is limited to statistical and epidemiological basics, was taught in the sixth semester. The demographic breakdown of the sample is summarized in table 1 (Tab. 1).

#### Measures

In order to determine if the students already have practical scientific experience, the following question was asked: “*Are you already carrying out scientific work or have you done so before? (for example as part of a doctoral thesis)”*. Scientific activity was deliberately not restricted to medical doctoral studies since there was a chance some students might have already gained comparable scientific experience in another degree. If ignored, this would have artificially reduced the potential effect of scientific experience in the analysis.

**Figure 1:**
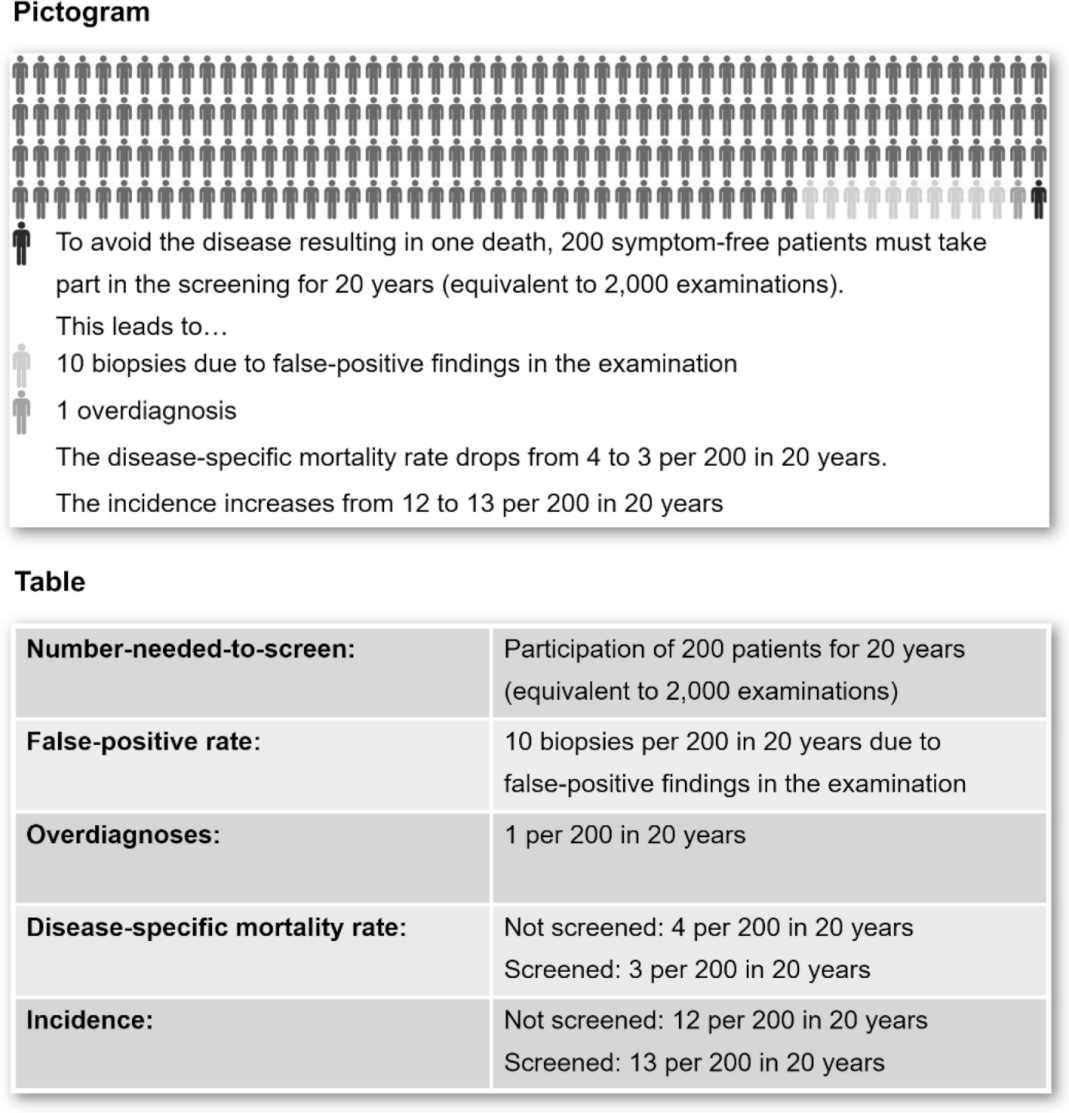
Screening scenarios: Presentation of epidemiological data as pictograms (upper part) and tables (lower part). Illustration created from screenshot of the questionnaire used.

Following Wegwarth et al. three decision-making scenarios were constructed [23]. The design of the scenarios corresponded to a previous study with GPs [24]. The epidemiological data on screening tests for the three most common types of cancer in Germany served as a basis: endoscopy for colorectal cancer screening, mammography for breast cancer screening and PSA-screening for prostate cancer [25], [26], [27], [28], [29]. Neither the diseases nor the screening tests were mentioned by name. This was to ensure that respondents’ decision-making was based solely on the figures from the evidence and detached from any preconceptions that might have increased their confidence. All scenarios started with the following introductory text: *“A 58 year old healthy patient with no relevant risk factors comes to your surgery and asks you for advice on a specific test for early detection of a tumor. The patient trusts your opinion and wants to know if you recommend this test to them. First, you research what evidence there is regarding risks and benefits and find the following information in reliable scientific studies.”* This was followed by figures on the incidence and mortality rate of the disease, as well as on Number-Needed-to-Screen (NNS) and potential harm (false-positive rate, complications, over-diagnoses). These figures were chosen as they are typically reported in studies on screening tests. In addition, all but the incidence are highly relevant for the evaluation. The figures were expressed as absolute risks and natural frequencies. Relative risks in the literature, if necessary, had been converted into these as well. The Number-Needed-to-Screen, where not already available in the literature, was calculated from the absolute risk reduction of the disease-specific mortality. Within the individual scenarios, care was taken to keep the denominators as uniform as possible. The format thus corresponds to the initially described recommendations for comprehensible presentation [16]. In addition, pictograms were created using Photoshop for the figures based on the recommendations for PSA-screening by DEGAM [29]. The pictograms and tables always contained the same figures with identical denominators. The wording in both presentations were only slightly different. Both forms of representation are shown in figure 1 (Fig. 1).

#### Questionnaire

Each respondent had to rate all three scenarios in random order once. The epidemiological figures for each scenario were randomly presented either as a pictogram or as a table of numbers. For example, a respondent might be presented with two scenarios with pictograms and one with a table. The randomization of the respondents to the different presentations of the scenarios as a pictogram or table of numbers resulted in approximately equal groups with a similar demographic composition. In each scenario, respondents were asked to make a recommendation on a 6-point Likert scale (I recommend the examination: 1=“definitely” to 6=“not at all”). The requested recommendations for the scenarios were meant to simulate an actual decision-making situation but were not part of the evaluation. Then the decision-making confidence had to be stated on a 6-point Likert scale (Regarding my confidence in my decision I am: 1=“very confident” to 6=“not confident at all”).

#### Data analysis

The evaluation was done using SPSS Statistics Version 21. All scenarios were analyzed individually and tested for a correlation between decision-making confidence and semester of study by means of product-moment correlations according to Pearson. For each respondent, the mean decision-making confidence in the three scenarios was calculated. A correlation between mean decision-making confidence and number of semesters was also tested using product-moment correlation according to Pearson. In addition, an exploratory analysis was conducted to find out whether students who have already attended the cross-curricular course on epidemiology and medical biometry show greater confidence in evidence-based decision-making. For this, the respondents who had not yet attended the course (semester 1-6) were compared with those who had already attended it (semester 7-11) by means of *t*-test for unpaired samples with regard to their average decision-making confidence. Subsequently, students who were scientifically active were compared with those who were not, using *t*-test for unpaired samples. In order to find out whether the decision-making confidence differs across all three scenarios, depending on the mode of presentation, a *t*-test for unpaired samples was also used.

## Results

### Decision-making confidence and semester

**Figure 2:**
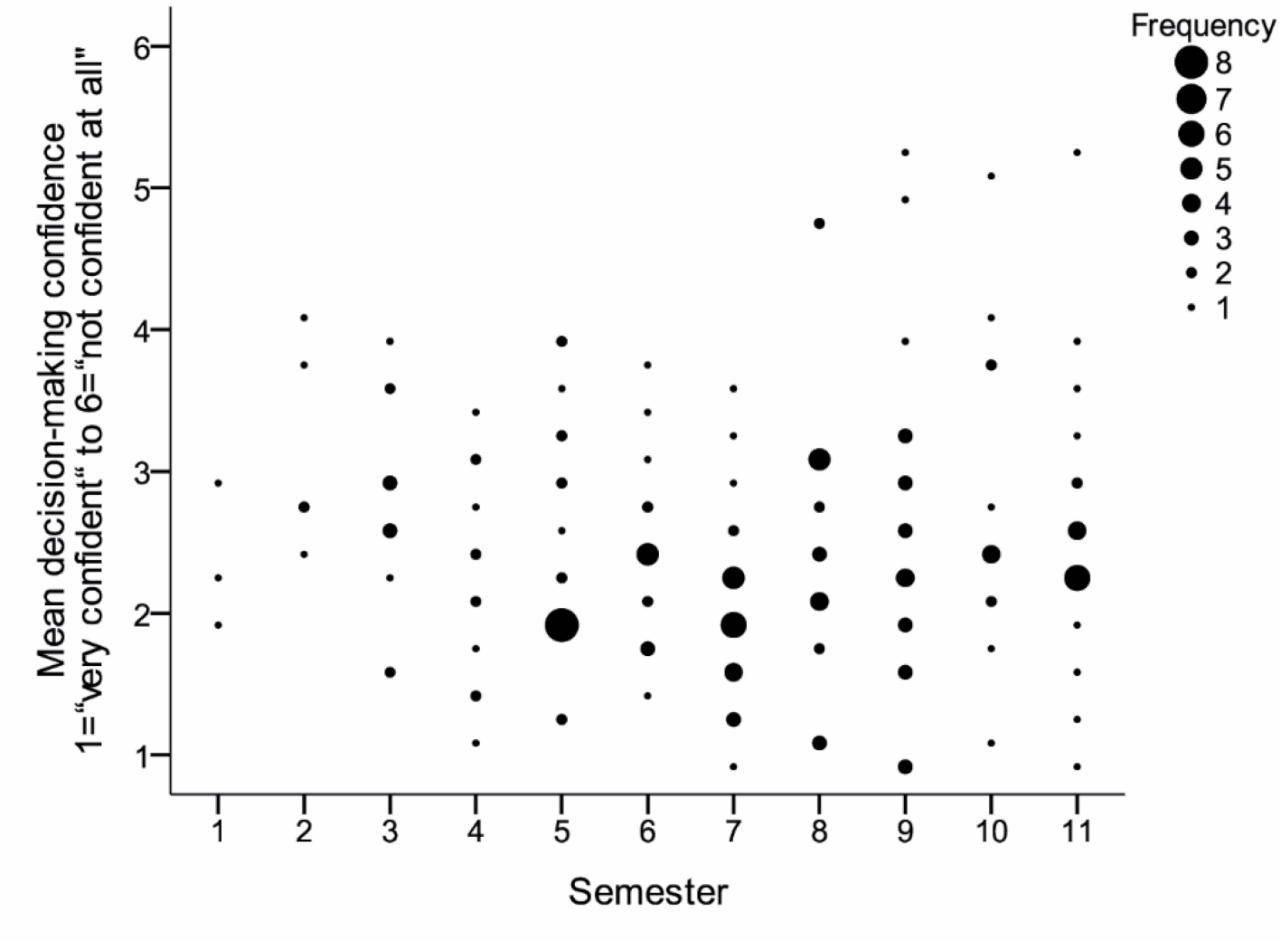
Scatter diagram of the mean decision-making confidence per respondent (*n*=171) in the three scenarios by semester. The size of the points reflects the frequency in cases of multiple values.

On average, the students were relatively confident in their decisions (*M*=2.48, *SD*=0.88). Decision-making confidence varied only slightly over the different semesters. A weak correlation between decision-making confidence and semester of study could only be demonstrated in one scenario (Scenario 1 Colorectal cancer screening: *r_Pearson_*=0.132, *p*=0.042, Scenario 2 Mammography: *r_Pearson_*=-0.043, *p*=0.29, Scenario 3 PSA-Screening: *r_Pearson_*=-0.045, *p*=0.28). Correspondingly, when considering the average decision-making confidence, there was no overall correlation in all scenarios (*r_Pearson_*=0.018, *p*=0.41). Attendance at the cross-curricular course on epidemiology and medical biometry also had no effect on the confidence of evidence-based decision-making (Course attended: *M*=2.47, *SD*=0.96, course not yet attended: *M*=2.50, *SD*=0.75, *t*(169)=-0.26, *p*=0.40, *d*=-0.04). An overview of the individual semesters can be found in table 2 (Tab. 2) regarding decision-making confidence in the scenarios and figure 2 (Fig. 2) for the mean decision-making confidence.

#### Decision-making confidence and scientific work

Students who are or have been involved in scientific work, for example as part of a doctoral thesis, were not significantly more confident in their decisions (with scientific work: *M*=2.38, *SD*=0.86, without scientific work: *M*=2.56, *SD*=0.90, *t*(169)=-1.26, *p*=0.11, *d*=-0.19).

#### Impact of the presentation

In the three scenarios (*n*=513), presentation as a pictogram led to significantly higher decision-making confidence amongst students than presentation as a table of numbers (pictogram: *M*=2.33, *SD*=1.07, table of numbers: *M*=2.64, *SD*=1.11, *t*(511)=3.21, *p*<0.01, *d*=0.28).

## Discussion

Overall, the respondents were relatively confident in their decisions based on epidemiological data. However, this decision-making confidence does not increase in higher semesters. Also, no connection was found between scientific work, for example as part of a doctoral thesis and higher confidence in clinical decision-making based on evidence. If the epidemiological data are presented graphically as pictograms, this increases decision-making confidence slightly.

Overall, students indicate that they are relatively confident in their decisions based on epidemiological data. It should be borne in mind that there may be bias due to social desirability of indicating higher decision-making confidence [30]. Despite these possible upward distortions, it is unexpected that decision-making confidence does not increase in higher semesters. Perhaps the low number of respondents in the pre-clinical semesters led to non-representative and overly high decision-making confidence being measured. However, when considering the clinical semesters alone, no increase in decision-making confidence was evident either. The reason for the lack of decision-making confidence could be that there is still too little evidence-based decision-making or critical appraisal of evidence in curricular courses and in clinical internships. The cross-curricular course on epidemiology and medical biometry cannot fill this gap. Students who have already attended such courses do not differ from their younger fellow students in their confidence in clinical evidence-based decision-making. This could be remedied by the, according to current surveys, steadily growing number of courses on evidence-based medicine in Germany [10]. However, according to the same study, these courses focus mainly on literature research and the subsequent review of studies. Transferring the evidence found to the patients and the associated decision-making was rarely the subject of the courses. The development of appropriate courses which feature this aspect should be pursued further so that medical students can learn confident clinical decision-making while at university.

In this study students who were already involved in scientific work, for example as part of a medical doctoral thesis, show no higher decision-making confidence when dealing with epidemiological data. This contradicts studies from the past. Medical students rated their scientific competences higher if they had already completed their doctorate [14], [15]. However, in past studies, medical students often overestimated their own ability to handle statistics [5], [6]. In addition, higher scientific competence does not necessarily go hand in hand with more confident clinical decision-making. The process of evidence-based decision-making is far more complex than the mere application of scientific skills because evidence often leaves considerable room for decision-making in clinical practice. Clinical decision-making for and with patients is not least a subjective decision and remains subject to considerable residual uncertainty. Perhaps that is why medical students feel that their scientific competence was strengthened by a doctorate but that they are no better prepared for their later work as a physician [15]. It is therefore questionable whether medical doctoral theses in their current form also adequately promote the application of scientific competencies to clinical questions. In order to reach a level of decision-making competence based on the application of scientific evidence in daily medical practice as expected by the NKLM at the time of graduation, a medical doctoral thesis on its own seems inadequate [http://www.nklm.de].

The way in which epidemiological data are presented influences, albeit to a lesser degree, the decision-making confidence of medical students. This fits with results from similar studies about understanding statistical data [19], [20]. For students of various subjects, presenting data in the form of pictograms improved comprehension of figures [20]. The effect was smaller the higher the already existing competence of the respondents was in dealing with figures. One would therefore also expect the medical students surveyed to have an improved understanding of the presented figures, which is associated with a higher level of confidence in dealing with them [7]. In another study, information presented in the form of pictograms was also found to be more helpful and trustworthy [19]. Digital decision-making aids for patients are increasingly common especially in general practice. Decision-making aids, such as the cardiovascular risk calculator arriba or the DEGAM recommendation for PSA-screening, not only support patients but also help doctors with evidence-based decision-making by graphically presenting data as pictograms [29], [31]. According to our findings, the use of such decision-making aids in medical education could provide medical students with extra confidence in clinical decision-making. Whether the results can be transferred to young doctors, however, is still unclear.

### Limitations

Limitations of the study are the low response rate, especially from the early semesters. How representative the individual results are is questionable here because of small group sizes in these semesters. However, it seems unlikely that this has caused a bias in the overall analysis. Another weakness can be found in the analysis of medical students with and without experience in scientific work. It was largely left open as to what kind of activity actually qualifies as scientific work. A doctorate was just mentioned as an example, so that scientific experience from another advanced degree could be taken into account. However, scientific activities of medical students also differ in their duration and intensity. But this heterogeneity of experience can hardly do justice to the simplicity of the operationalization used. As a result, a connection between decision-making confidence and scientific work may have been diluted and overlooked. In addition, students with little affinity to statistics were less likely to participate in the study. Social desirability may also have resulted in higher decision-making confidence being indicated [30]. These characteristics of the sample may have led to an overall overestimation of decision-making confidence. Nonetheless, the relative differences between the sub-groups examined should not have been biased by this. Since this is a cross-sectional study, it is not possible to draw clear conclusions about the effects of time on decision-making confidence over the course of study. Only speculations can be made based on the relationships found. In order to investigate the change in decision-making confidence over the various semesters or through scientific work without bias, longitudinal studies are necessary. How representative the results are for other faculties is limited because the study was conducted at only one faculty.

## Conclusions

Compared to lower semesters, contrary to expectations medical students from higher semesters do not show higher decision-making confidence. It is questionable whether they gain additional confidence in evidence-based clinical decision-making as part of the curriculum. Scientific work, such as a doctoral thesis, does not seem to strengthen the required skills sufficiently. However, this study can only speculate. To confirm these hypotheses, additional longitudinal studies are necessary. Nonetheless, medical education should put more emphasis on evidence-based medicine and, in particular, clinical decision-making based on evidence. Additionally, clinical decision aids using pictograms can give students confidence in decision-making. 

## Declarations

This study was conducted without specific financial support from public, commercial or not-for-profit entities. The present work is performed in partial fulfillment of the requirements for the degree “Dr. med” at the Friedrich-Alexander-University Erlangen-Nürnberg.

All research performed was consistent with the ethical standards of the Helsinki Declaration (Recast Fortaleza 2013) and the Geneva Declaration. The clearance certificate of the Ethics Commission of the Medical Faculty of the Friedrich-Alexander-University Erlangen-Nürnberg was issued (327_14c from 14.11.2014).

## Competing interests

The authors declare that they have no competing interests. 

## Figures and Tables

**Table 1:**
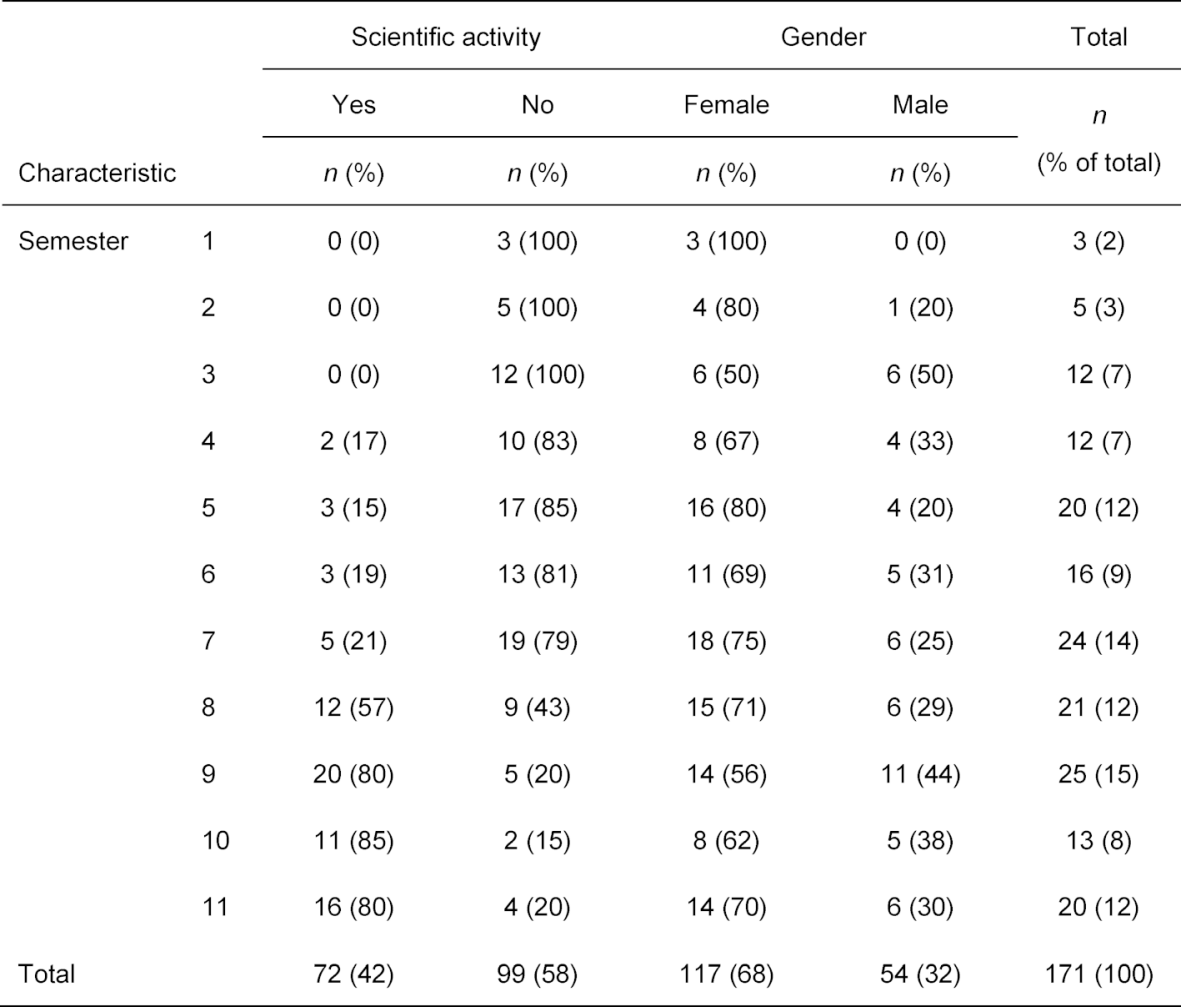
Demographic characteristics of respondents (*n*=171) by semester. Unless otherwise stated, the frequencies (with the percentages in parentheses) are given in relation to each line.

**Table 2:**
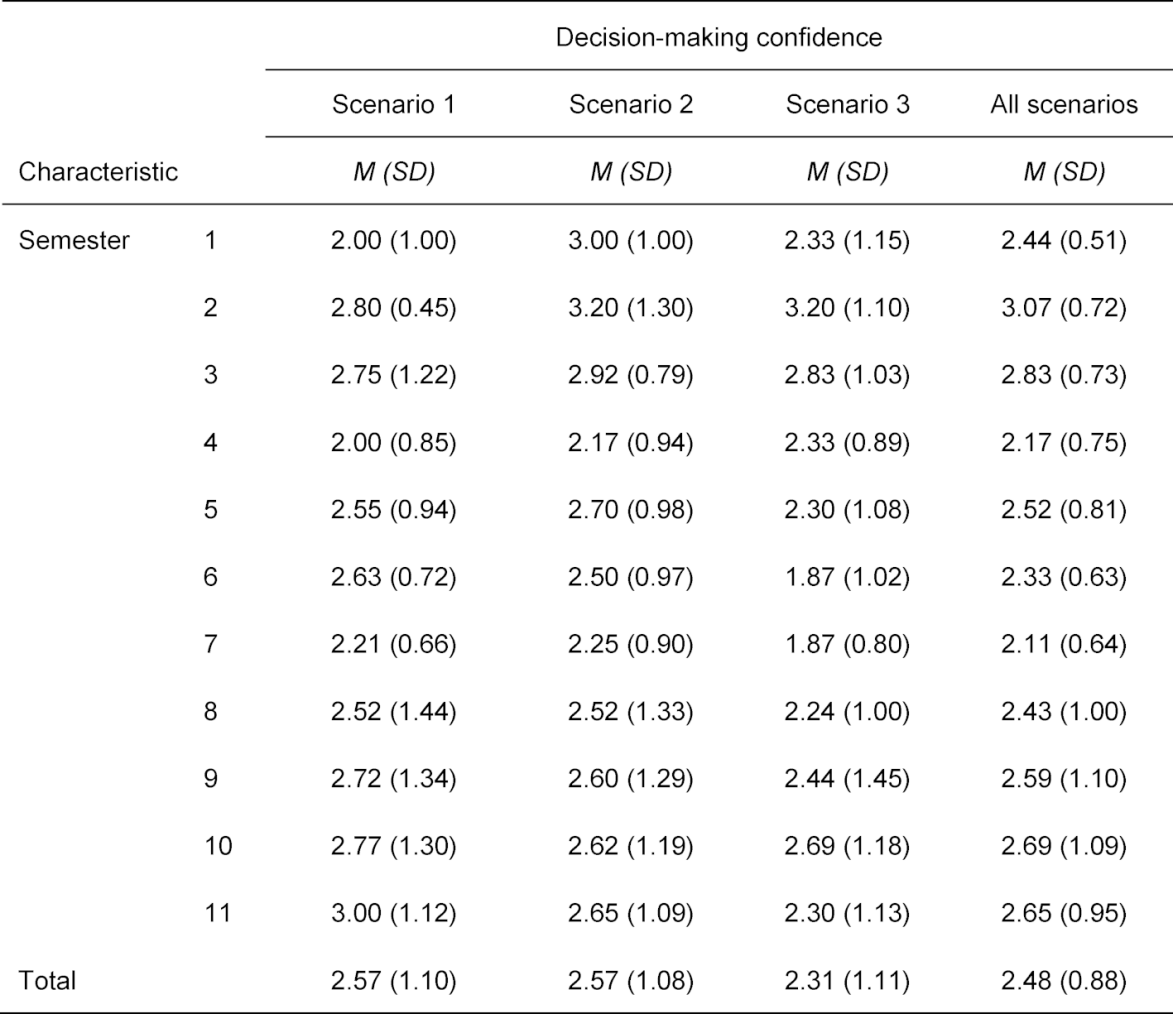
Mean decision-making confidence and decision-making confidence in the three scenarios of the respondents (*n*=171) by semester. Scenario 1 used the data for colorectal cancer screening, Scenario 2 the data for mammography and Scenario 3 the data for PSA screening.
